# Evaluation of a model of online, facilitated, peer group supervision for dietitians working in eating disorders

**DOI:** 10.1186/s40337-022-00617-7

**Published:** 2022-07-04

**Authors:** Amanda Davis, Nina Meloncelli, Amy Hannigan, Warren Ward

**Affiliations:** 1 Queensland Eating Disorders Service, Metro North Hospital and Health Service, Brisbane, Australia; 2 Royal Brisbane and Women’s Hospital, Allied Health, Metro North Hospital and Health Service, Brisbane, Australia

**Keywords:** Online peer group supervision, Dietitian, Eating disorders, Credentialing, Practice standards, Clinical supervision, ANZAED

## Abstract

**Background:**

The recently published *Australia and New Zealand Academy of Eating Disorders* (ANZAED) *practice and training standards for dietitians providing eating disorder treatment* recommended dietitians working in eating disorders (EDs) seek further clinical experience, training, and supervision to provide effective evidence-informed treatment. Access to dietetic clinical supervision is problematic, secondary to limited trained supervisors, location, cost, and lack of organizational support. Demand for clinical supervision increased with the 2022 introduction of ANZAED credentialing for eating disorder (ED) clinicians in Australia and addition of the Eating Disorder Management Plan to the Medicare Benefits Scheme. In 2018, QuEDS piloted a model of online peer group supervision with the goal of increasing service capacity to provide ED-specific clinical supervision to dietitians. Positive evaluation of the pilot led to the rollout of QuEDS Facilitated Peer Supervision (QuEDS FPS) program which was evaluated for utility and acceptability.

**Methods:**

By August 2021 five QuEDS FPS groups were established each with a maximum of 10 Queensland-based dietitians from public hospital, community, or private practice plus an additional Facilitator and Co-facilitator. A total of 76 participants enrolled in the program over the study period in addition to the 10 participants from the pilot program. Participant experience was evaluated with anonymous, voluntary surveys at baseline (59 responses), 6 months follow-up (37 responses), plus a one-off survey in August 2021 (50 responses). Pilot participant’s Baseline and Follow-up surveys were not included in this evaluation.

**Results:**

Survey responses were positive across the four Kirkpatrick training evaluation domains of reaction, learning, behavior, and results. Respondents reported positive change to clinical practice (98%), including increased confidence to implement evidence-informed guidelines, and improved engagement with, and advocacy for, ED clients. Service capacity to provide supervision was increased by high participant to Facilitator ratios (10 participants to one Facilitator and one Co-facilitator) and recruitment of external Facilitators. Respondents indicated they would recommend QuEDS FPS to other dietitians and 96% planned to continue with the program.

**Conclusions:**

QuEDS FPS program increases capacity to provide supervision with demonstrated positive impacts on dietitians’ confidence and ability to deliver dietetic interventions in the ED arena and, by inference, the dietetic care of people with an ED.

**Supplementary Information:**

The online version contains supplementary material available at 10.1186/s40337-022-00617-7.

## Plain English summary

Dietitians often lack confidence and specialist skills to provide best practice care for people with eating disorders. Recent initiatives to improve access to dietetic treatment for people with an eating disorder in Australia have increased demand for eating disorder-specific clinical supervision for dietitians. To address demand for supervision with limited resources, the Queensland Eating Disorder Service developed a new model of peer group supervision. This model consisted of monthly 90 min online groups of 10 dietitians with a Facilitator and Co-facilitator. The sessions were facilitated using a script and formats to ensure all participants could contribute and be supported equally. Only principles of best practice were propagated. Participants reported an increase in clinical confidence and skills to deliver eating disorder-specific dietetic interventions and satisfaction in this important work, plus in their ability to advocate for most appropriate care for people with eating disorders. This model increases service capacity to provide effective supervision to large numbers of dietitians through high ratios of participants to supervisors.

**Supplementary Information:**

The online version contains supplementary material available at 10.1186/s40337-022-00617-7.

## Background

Provision of eating disorder (ED) treatment in Australia is challenging secondary to lack of access to specialist ED services, which are traditionally based in metropolitan hubs [1]. Clinicians in generalist services provide first point-of-care, especially in rural and remote areas [1]. Limited undergraduate training of health professionals (including dietitians) in ED treatment has been identified as a workforce challenge [2] and lack of clinical experience, ‘especially in the area of ED management principles, may cause unintentional harm or even delay recovery’ [2].

Recent initiatives to improve the quality and access to treatment for people with EDs in Australia include the publication of the *Australia New Zealand Academy of Eating Disorders (ANZAED) eating disorder treatment principles and general clinical practice and training standards* [3], the 2021–2022 implementation of ANZAED’s credentialing system for clinicians [4] and the introduction of Medicare-funded Eating Disorder Management Plans. One effect of these initiatives has been an upsurge of interest in clinical supervision for clinicians working in the ED arena. ANZAED credentialing standards include specified levels of training, ongoing professional development and planned clinical supervision [3].

The ‘*ANZAED Practice and Training Standards for Dietitians providing eating disorder treatment’* identified gaps in training for dietitians working in this field [5]. Participation in clinical supervision has a role in upskilling practitioners in ED-specific treatment [6] and positively impact ED treatment outcomes [7]. The Queensland Eating Disorder Service (QuEDS) provides training, consultation, and advice to clinicians. Previous dietitian-specific support has been provided via individual supervision, ad hoc peer-to-peer mentoring, targeted education, and creation of dietetic peer networks. However, these strategies are reliant on QuEDS’ limited resources and expertise and the growing demand for supervision became unsustainable.

A 2017 QuEDS’ survey of dietitians from nine Queensland hospitals found that although respondents felt ‘well-supported’ by QuEDS, the majority requested further educational opportunities, especially options such as on-line training and webinars. (Report *Quality Activity—Queensland Eating Disorder Service QuEDS Consultation Service. Improving quality of QuEDS-CS to public hospital dietitians*. Available on request from author). QuEDS consultation service receives more requests from dietitians seeking ED-specific support and training than can be supported with individual supervision. However, there is a dearth of studies available on the effectiveness of group clinical supervision for allied health [8].

The familiarity of the New Zealand Coaching and Mentoring Centre Peer Group Supervision model within Queensland Health provided the impetus to explore a modified format of group supervision to better suit the ED context and to meet the demand for high-quality ED-specific dietetic supervision [9]. In 2018, QuEDS conducted and evaluated a 12 month pilot of an innovative model of online group supervision for Queensland Health dietitians [10]. The QuEDS Peer Group Pilot model was congruent with the definition of supervision as a ‘formal activity for professional development and learning where there is an emphasis on discussion, feedback, guidance and support with the aim of enhancing the functionality, quality and capability or effectiveness of the supervisee’ [11]. In addition, principles of adult learning theory (self-directed, acknowledged experience and knowledge, goal-oriented, relevant, practical, respectful) were utilized in development of the QuEDS Peer Group Pilot format [12].

QuEDS Peer Group Pilot differed from the usual model of Peer Group Supervision for Queensland Health allied health professionals*—*its online format enabled access for dietitians outside of metropolitan areas and encouraged mixed groups with respect to experience levels, locations and workplaces to promote more open sharing of experiences. It was structured to ensure all participants’ equal opportunity to participate and be supported, and facilitated to ensure only safe, evidence-informed practice was propagated, fidelity to the model, adherence to time allocations, and identification of in-session learning opportunities. The positive evaluation of the 2018–2019 pilot group informed the roll out of the QuEDS Facilitated Peer Supervision (QuEDS FPS) program for dietitians working with ED clients, including access for private dietetic practitioners.

Here, we provide an evaluation of the QuEDS FPS program, following the pilot. Emphasis is on utility, acceptability, and sustainability as a cost-effective model of provision of clinical support to large numbers of clinicians working in the ED field. The QuEDS FPS documentation suite and implementation process is openly available online to encourage replicability and comparison with other models [13].

## Methods

### An overview of the QuEDS Facilitated Peer Supervision (QuEDS FPS) Model

Full details of the QuEDS FPS model and its implementation are described elsewhere and publicly available online [13]. A brief overview is provided here. Following the pilot QuEDS Peer Group program, the QuEDS FPS program was launched with an additional four groups. QuEDS FPS model consisted of online monthly 90 min group sessions, each of 10 participants, with an additional allocated Facilitator and Co-Facilitator. Details of the groups, including clinical practice areas are described in Table 1.

**Table 1:** Characteristics of survey respondents for the Queensland Eating Disorder Service Facilitated Peer Supervision groups

	Baseline survey	Follow-up survey (6 months)	Learning and clinical practice survey
Total respondents	59 (78%)	37 (56%)	50 (71%)
Facilitated Peer Supervision groups^a^ (plus clinical practice area)			
A (general focus) n = 19 (includes 10 pilot participants)	7 (12%)	3 (8%)	11 (22%)
B (private practice) n = 23	16 (27%)	9 (24%)	13 (26%)
C (paediatric/adolescent focus) n = 20	16 (27%)	8 (22%)	8 (16%)
D (private practice) n = 13	11 (19%)	6 (16%)	10 (20%)
E (community focus) n = 11	6 (10%)	4 (11%)	8 (16%)
Not stated	3 (5%)	7 (19%)	
Location			
Metro (urban centre population > 100,000)	22 (37%)	13 (35%)	
Capital city (Brisbane)	21 (36%)	15 (41%)	
Rural (urban centre population10,000–99,999 population)	12 (20%)	7 (19%)	
Remote (urban centre/area population < 9,999)	3 (5.1%)	2 (5.4%)	
Not stated	1 (1.7%)		
Experience			
< 5 years	27 (46%)	20 (54%)	
5–10 years	19 (32%)	8 (22%)	
> 10 years	13 (22%)	9 (24%)	
Number of clients (past 12 months)			
< 5 clients	21 (36%)	10 (27%)	
5–15 clients	21 (36%)	13 (35%)	
> 15 clients	17 (29%)	14 (38%)	
Place of employment			
Public hospital	24 (41%)	17 (46%)	
Public Community Health Centre	8 (14%)	9 (24%)	
Private hospital	8 (14%)	3 (8.1%)	
Private practice	30 (51%)	18 (49%)	
University clinic	2 (3%)	1 (2.7%)	
Public specialist eating disorder service	1 (2.7%)	0 (0%)	
Non-government organization	4 (7%)	1 (2.7%)	
Dietetic student	1 (2.7%)		
Client group			
Pediatric	20 (34%)	8 (22%)	
Adolescent	46 (78%)	27 (73%)	
Adult	50 (85%)	35 (95%)	

Percentages for Baseline and Follow-up Surveys are based on all participants not including the original pilot group (n = 76). Percentages for the Learning and Clinical Practice Survey based on 70 past and present participants with a valid email (including pilot participants)

^a^ Groups included 10 participants at any one time. Higher numbers for each group reflect withdrawals and subsequent inclusion of additional members from the waitlist

The QuEDS Lead Facilitator, who developed the model, was responsible for overarching administration of the QuEDS FPS program including Facilitator training, mentoring and program evaluation. Facilitators were recruited and orientated by the Lead Facilitator and were expected to have more than 5 years clinical experience within the peer supervision group’s clinical practice area (e.g., > 5 years’ experience in dietetic intervention for paediatric and adolescent clients, for the paediatric/adolescent ED clinical practice group) and participated in QuEDS FPS for at least 6 months. Co-facilitators were considered Facilitators-in-training, provided back-up to the Facilitator role, and provided for program expansion. Essential skills included understanding of the application of professional boundaries and risk management for the clinical practice area. Sessions were facilitated using the QuEDS FPS model session format and script and documented as a session summary to be emailed to participants [13]. Facilitators avoided the role of ‘expert’ within the group, whilst ensuring best practice was propagated through participants sharing experience and, if necessary, with guided discussions to achieve this.

QuEDS FPS program consisted of one Lead Facilitator for the program, 5 ongoing groups, each with a maximum of 10 participants, plus one Facilitator and one Co-facilitator allocated per group, providing monthly sessions for a total of more than 50 participants at any time. Participation in the groups was invited through expression of interest documents which were distributed throughout Queensland.

Participants were eligible to apply if they were a Queensland-based Accredited Practicing Dietitian, in either the public health or private domain, with an interest in treating, or currently treating people with ED diagnoses, and the ability to commit to attendance of at least 10 out of 12 sessions annually. Applications were accepted on a first-come, first-served basis. Unsuccessful applicants were added to the QuEDS FPS waitlist.

### Evaluation

Participants (excluding those who had taken part in the pilot program) were invited to complete surveys at pre-commencement (Baseline) (see Additional file 1) and Follow-up at 6 months (see Additional file 2). Survey links for pre-commencement (Baseline) and 6 month (Follow-up) surveys were emailed to current participants at time points as triggered by their start dates. Surveys were anonymous and voluntary. Linkage between surveys was attempted through generation of a personalized code. Participants who withdrew from the QuEDS FPS program prior to 6 months were not invited to complete the Follow-up survey. Surveys were designed to evaluate the acceptability and utility of the model. To identify the impact of QuEDS FPS participation on changes to clinical practice, a decision was made to conduct a Learning and Clinical Practice Survey in August 2021 (see Additional file 3). All participants (including withdrawals) from the initiation of the program in 2018 until August 2021 were invited to respond to the Learning and Clinical Practice Survey.

For purposes of evaluation, the Baseline, Follow-up, and Learning and Clinical Practice Surveys were compared.

Baseline and Follow-up surveys were developed with reference to the Clinical Supervision Evaluation Questionnaire (CSEQ) [14], the Metro North Hospital and Health Service Peer Supervision Group Evaluation form (informed by New Zealand Coaching and Mentoring Centre) [9], previous surveys from the QuEDS Peer Group Supervision pilot program and the Kirkpatrick Model for Training evaluation [15]. An overview of survey development is available online [13] which includes relevant Kirkpatrick levels of training evaluation [15]. The Learning and Clinical Practice survey was informed by the four-area Kirkpatrick training evaluation model and designed to capture additional data on self-assessed change in clinical practice directly attributed to participation in QuEDS FPS program.

Implementation of the program was evaluated using the RE-AIM framework [16] and described elsewhere [13].

### Data analysis

Results analyses were conducted by a third party who did not take any part in the program. Results from each survey (Baseline, Follow-up, and Learning and Clinical Practice surveys) were reported descriptively under the relevant Kirkpatrick Level where applicable. Continuous data was reported as mean (standard deviation), while categorical data was reported as *n* (%). The questions from the surveys that relate to each Kirkpatrick level are shown in detail in Additional file 4.

Not all Baseline and Follow-up surveys were able to be linked (for example: inconsistent personalized codes, different respondents to each survey). Therefore, the pooled results were reported for each survey, and no attempt was made to infer within-person changes to any of the Kirkpatrick Levels. However, to understand the mean change in confidence ranking (three questions) between Baseline and Follow-up surveys for linked responses that were able to be determined, a regression model was used to examine the association between confidence and survey time (Baseline and Follow-up). A mixed model with a random intercept per person was utilized to account for the expected within-person correlation. We used unadjusted and adjusted models (years’ experience, numbers of patients seen and location) to determine the differences in mean confidence (95% confidence interval). As the adjusted and unadjusted results were similar, only the results of the unadjusted models have been provided. The adequacy of the normal assumption was tested by plotting the model residuals, which provided strong evidence that there was no need to use non-parametric statistics.

## Results

Group A (original pilot group) transitioned as a ‘business as usual’ group from May 2019. Groups B and C commenced in September 2019 and Groups D and E commenced in January 2021.

There were 76 total new recruits to the program following the pilot group and all groups have functioned at 100% capacity (10 participants + 2 Facilitator/Co-facilitators) since inception, except for Group E due to relatively lower numbers of dietitians working in community mental health positions in Queensland. Recruitment into vacancies arising secondary to withdrawals is reflected in the higher total group numbers shown in Table 1.

There were 27 withdrawals from the QuEDS FPS program during the evaluation period between May 2019 and August 2021. Ten participants withdrew prior to six months' participation and did not complete the Follow-up Survey. Nine participants withdrew secondary to parental leave and 18 due to workload or job change.

The Baseline Survey was sent to all participants (excepting the 10 participants from the pilot group) and completed by 59 of the 76 total participants (77.6% response rate). The Follow-up Survey was completed by 37 of the 66 possible participants (56% response rate). The Learning and Clinical Practice Survey was sent to all past and current participants (including pilot group) with valid email addresses (70 of 86) and received 50 responses (71% response rate). Participant characteristics are shown in Table 1 for all three surveys.

Of the Follow-up Survey respondents, 28 (76%) could be matched to the Baseline Survey. The Learning and Clinical Practice Survey was not intended to be matched to previous surveys. A greater proportion of the total responses to the Baseline and Follow up Survey were from Group B and C participants, reflecting the larger number of participants through these groups (longer duration than Groups D and E) and the exclusion of pilot participants (original participants of Group A) from the baseline and follow-up surveys. Almost three quarters of respondents (73% baseline, 76% follow-up) were from a Metro center or capital city (Brisbane) (see Table 1). More than 20% of participants were from rural and remote centers. Around half the respondents in both groups (46% and 54%) reported having less than 5 years clinical experience, with more than 20% greater than 10 years' experience (Table 1).

### Kirkpatrick level 1: reaction

Most participants in QuEDS FPS program reported positive experiences across all *Reaction* domains Additional file 4. Participants reported reaction to the QuEDS FPS process included high levels of feeling ‘safe’ (95%), maintenance of ‘confidentiality’ (100%), positive feelings of ‘confidence’ (100%), feeling ‘supported’ (95%) and positive impact of group facilitation (97%). Ninety-six percent of respondents reported their intention to continue to participate. Participant’s reactions to the QuEDS FPS model (in the follow-up survey) with respect to preferred mode of upskilling in dietetic intervention for EDs were ranked from most to least preferred as follows: Individual supervision, QuEDS FPS, specialist education sessions, workshops, guidelines, peer group supervision, online education modules. Ninety-two percent of respondents in the Learning and Clinical Practice survey preferred the QuEDS FPS format to Peer Group Supervision model utilized by Queensland Health Allied Health leadership [9].

### Kirkpatrick level 2: learning—self-assessed increase in skills

Learning was assessed in the follow-up survey and the Learning and Clinical Practice survey. For most participants, individual learning expectations were met across all domains (see Additional file 5). From the Learning and Clinical Practice survey, of the 50 respondents, the number of respondents reporting unmet learning expectations (from highest to lowest) are as follows: ED-specific counselling skills (n = 6 of 35); ED-specific evidence-based practice/guidelines (n = 4 of 28); assessment/treatment of ED diagnoses (n = 4 of 30); ED-specific tools/resources (n = 3 of 43); Complex ED case management (n = 2 of 28); ED presentations (n = 1 of 29); confidence to implement ED-specific dietetic interventions (n = 1 of 41).

#### Changes in self-assessed confidence

Three questions in the Follow-up survey could be assessed as pre-post changes to ratings. There was a similar increase in confidence for the adjusted and unadjusted models, therefore, only the results of the unadjusted regression modelling are provided. The mean change in confidence (95% CI) from Baseline to Follow-up Surveys was consistent across all three questions.

For the statement ‘*I feel confident applying evidence-based practice in the treatment of eating disorders’* the mean confidence rating was 0.7 (0.4 to 0.9) higher in the Follow-up survey. For the statement *‘I feel confidence engaging/communicating with people with eating disorders’* (communication), the mean confidence was 0.6 (0.4 to 0.9) higher, and for the statement *‘I feel supported as a dietitian working in the field of eating disorders’*, the mean confidence was 0.9 (0.6 to 1.2) higher in the Follow-up survey.

### Kirkpatrick level 3: behavior—implementation of newly acquired skills/knowledge

Most respondents (88%) felt that they had changed their clinical practice by improved implementation of evidence-based guidelines and (90%) application of ED-specific tools and resources (Additional file 4). Participants (94%) also felt they had increased their ability to provide dietetic intervention for complex ED cases, and 88% agreed that participation in QuEDS FPS program had increased their reflective practice (Additional file 4).

### Kirkpatrick level 4: results—broader impacts of participation in QuEDS FPS program

Participation in QuEDS FPS groups impacted on self-reported results across several domains. Respondents reported increased ‘confidence’ (96%) in clinical work, improved ‘engagement’ (88%) with, and increased ‘advocacy for appropriate care’ (90%) for ED clients (Additional file 4).

Ninety-eight percent of respondents reported participation in QuEDS FPS enabled them to feel ‘supported’ in their clinical work, to cope better with ‘stressors of working with ED clients’ (86%), and to better ‘enjoy work in the ED arena’ (84%). Ninety-four percent felt QuEDS FPS had helped them to ‘achieve more’ and eighty percent (80%) felt more actively engaged in ‘service development’ in the ED arena.

### QuEDS FPS program implementation informed by the RE-AIM framework

Detailed documentation of sessions in addition to the surveys, assisted in evaluating the implementation of the QuEDS FPS program using the RE-AIM framework [16]. Table 2 provides a breakdown of the RE-AIM parameters. Reach of the program was demonstrated by drawing participants from 11 of the possible 16 Queensland Hospital and Health Service areas, with more than 25% of participants from rural and remote areas, and ~ 50% of participants involved in private dietetic practice.

**Table 2:** Evaluation of the Queensland Eating Disorder Service Facilitated Peer Supervision Program according to the RE-AIM framework [16]

RE-AIM domain	Measure	Result
Reach^a^	Participant characteristics	11/16 possible Queensland Hospital Health Services represented > 25% rural and remote clinicians ~ 50% private practitioners
Efficacy^b^	Impact on: Clinician, Patient, Service outcomes	94% clinicians increase ability to provide dietetic interventions 90% increased advocacy for client care 94% increased involvement in ED-specific service development activities
Adoption	Uptake by other services, group**s**	Not yet demonstrated*—*interest from other services
Implementation^a^	Fidelity to model Cost to deliver	Not measured*—*ensured by scripting and Lead Facilitator role in supporting Facilitators ~ 22.5 h Lead Facilitator & Facilitator/co-Facilitators provides ~ 75 h participant support
Maintenance	Participant: Recruitment^a^ Retention^a^ Engagement^b^	86 recruits April 2018 to August 2021 27 withdrawals 9 parental leave 18 workload/position change 71% respondents to voluntary Learning and Clinical Practice survey 96% intention to continue^b^

Evaluation of program implementation used data from ^a^FPS program administration records and ^b^Baseline, Follow-up and Learning and Clinical Practice surveys

Efficacy or impact of the program on participants, as derived from the self-assessed surveys, suggested positive outcomes (> 90%) of self-assessed increased ability to provide dietetic interventions, advocacy for client care and involvement in ED-specific service development activities. Direct patient and service outcomes were not measured. Uptake by other services was not measured by this study. Implementation costs measured in clinician time were as follows: total Facilitator time (Lead Facilitator time, plus group Facilitator time, plus Co-facilitator time) of ~ 22.5 h per month provided 5 groups of 10 participants with 90 min per month of clinical support. Program maintenance was demonstrated by participant retention as documented at time of withdrawal from the program. There were 27 withdrawals over the study period. The majority of withdrawals (N = 18) were secondary to workload/position changes, with 9 participants taking parental leave. Strong participant engagement was determined by 96% intention to continue with QuEDS FPS program and high proportion of participants engaging in voluntary surveys (Learning and Clinical Practice Survey—71%).

## Discussion

The purpose of the current study was to evaluate the effectiveness of QuEDS FPS model which was developed to increase QuEDS’ supervisory capacity and enable broader access to cost-effective, appropriate clinical supervision for dietitians working in the ED arena. The utility, acceptability and impact of the model were evaluated according to the four Kirkpatrick Levels for training evaluation [15]. Participant responses to the evaluation surveys were uniformly positive across the four Kirkpatrick Levels of reaction, learning, behavior, and results.

Recent significant advances to improve access to evidence-informed dietetic interventions for people with eating disorders in Australia include the *ANZAED clinical practice and training standards for dietitians* [5], the introduction of ANZAED credentialing for eating disorder clinicians [4], and the addition of the Eating Disorder Management Plan to the Medicare Benefit Scheme [2]. These initiatives increased demand for ED-specific dietetic supervision to meet stated requirements for appropriate clinical practice [4, 5] and through increased numbers of clinicians providing ED treatment. This study is important because it evaluated a new peer group supervision model that increased service capacity to provide clinical dietetic supervision to help meet increased demand. Valuable findings were that the QuEDS FPS program delivered effective, accessible, acceptable clinical supervision to large numbers of dietitians over a large geographical area and across public and private arenas. Supervisory capacity was increased through high participant to supervisor ratios (10 participants to one Facilitator and Co-facilitator), recruitment of Facilitators external to QuEDS, and model replicability secondary to the documentation suite and centralized administration of the program. Reach was achieved through use of an online platform, organizational support from QuEDS and the dietetic leadership in Queensland Health, and no cost for participation. Fidelity to the model was achieved via a documentation suite including session format and script, non-rotating Facilitators, centralized administration, and support from a Lead Facilitator including orientation of participants and support to Facilitators. Sustainability to date has been made possible due to ongoing organizational support and use of Co-facilitator positions as Facilitators-in-training, plus strong participant engagement with 96% of participants indicating their intention to continue.

The *ANZAED clinical practice standards* state ‘clinical supervision and ongoing professional development aim to upskill clinicians, support reflective practice, aid the provision of high-quality treatment, and recognise the intensity and personal impact of treating complex mental health issues’ [3]. This evaluation showed QuEDS FPS program outcomes aligned with these aims as evidenced by participants’ self-assessed increased confidence to implement evidence-based dietetic care, increase in reflective practice, and improved feelings of enjoyment and decrease in stress associated with the ED workload. Ninety-eight percent of participants reported that their participation positively impacted their clinical practice and the majority of respondents felt QuEDS FPS had helped them to ‘achieve more’, be more actively engaged in ‘service development’ in the ED arena, and more confidently implement ‘best practice’ and advocate for clients. The QuEDS FPS was rated second only to individual supervision (50%) as a preferred mode of clinical upskilling. This differs markedly from Denman et al. [2] who reported preferred upskilling for dietitians working in the ED arena in the following order: online webinars, workshops, online courses, followed by clinical supervision, work shadowing and professional interest groups.

Accessibility to online clinical support and supervision has become increasingly important since the COVID-19 pandemic but concerns as to its effectiveness must be addressed. Accessibility to clinical supervision for rural and remote clinicians, isolated both geographically and collegiately, is important to ensure appropriate clinical care. Collegiate isolation is also common within the private practice arena. Private practitioners (often newly graduated) are increasingly likely to encounter clients with EDs in the community secondary to recent government incentives via the Medicare Benefit Scheme Eating Disorder Management Plan [2]. QuEDS FPS program was delivered online to increase accessibility for rural/regional clinicians and to enable a broad mix of participation in each group. QuEDS FPS program provided support to rural and remote clinicians, who accounted for ~ 25% of participants, and private practitioners (~ 50% participants) and was enabled by online delivery at zero cost to participants. QuEDS FPS survey respondents indicated they would recommend the program to other dietitians and 96% planned to continue with the program, a positive indicator of program sustainability. This study provided evidence that an entirely online model of peer supervision is acceptable and effective across both public and private domains.

The strength of this study is its appraisal of the QuEDS FPS model across the four Kirkpatrick levels for training evaluation and the Lead Facilitator’s role in assuring fidelity in delivery of the evaluated model. Inclusion of a Baseline survey in addition to the Follow up survey enabled measurement of significant positive changes in confidence to deliver ‘evidence-based care’ and to ‘engage with clients’ over the first six months of participation in QuEDS FPS. The study had high response rates across all surveys, which decreased selection bias.

Limitations in this study include the use of self-assessment measures in the surveys rather than direct measurement of clinical practice changes, impacts on patient or service outcomes, and limited participant numbers. Of the twenty-seven (27 of 76) withdrawals over the evaluation period, ten of these were prior to the Follow-up survey at 6 months, which they were not asked to complete. All withdrawals were secondary to job changes or parental leave. To address possible bias all past and current participants were invited to complete the Learning and Clinical Practice survey. This study used surveys which were not validated but were informed by CSEQ and the Kirkpatrick 4 Level Training Evaluation framework. This was a considered decision by the authors to fully consider data collection that could influence future refinements to the QuEDS FPS model.

## Conclusions

This study demonstrated a positive evaluation of an innovative model of online peer group supervision which increases capacity to provide supervision to dietitians working in the ED arena. QuEDS FPS program is an effective, accessible, online model of peer group supervision that supports dietitians working to improve clinical skills, increase confidence and take a more active role in advocacy for optimal ED patient care and in ED-specific service development with inferred broader positive impacts on the dietetic ED workforce, organizations and the dietetic care of people with eating disorders. In view of the positive evaluation of the QuEDS FPS program and its outcomes, which align with the *ANZAED clinical practice standards* stated aims for clinical supervision, it is possible to conclude that QuEDS FPS model of peer group supervision is an appropriate adjunct, or alternative, for individual clinical supervision and can be utilized to increase service capacity to provide effective supervision.

## Supplementary Information

**Additional file 1.** Baseline survey. Pre-commencement survey questionnaire.

**Additional file 2.** Follow-up survey. 6 month follow-up questionnaire.

**Additional file 3.** Learning and clinical practice survey. Questionnaire to determine clinical practice change secondary to participation in FPS.

**Additional file 4.** Responses in Kirkpatrick levels. Responses according to the four levels for Kirkpatrick’s Model of Evaluation.

**Additional file 5.** Learning expectations. Participants learning expectations of QuEDS FPS and unmet expectations.

## Data Availability

The datasets generated and analyzed during this study are available from the corresponding author on reasonable request.

## Abbreviations

ANZAED: Australia and New Zealand Academy of Eating Disorders
ED: Eating Disorder
EDs: Eating Disorders
QuEDS: Queensland Eating Disorder Service
QuEDS FPS: QuEDS Facilitated Peer Supervision
CSEQ: Clinical Supervision Evaluation Questionnaire
RE-AIM: Reach, Efficacy, Adoption, Implementation, Maintenance
