# Supplementary material for: Evaluation of a model of online, facilitated, peer group supervision for dietitians working in eating disorders
Source: J Eat Disord. 2022 Jul 4;10:93. doi: 10.1186/s40337-022-00617-7 (PMC9252553; doi:10.1186/s40337-022-00617-7)
Supplement: Supplementary file 1 — Additional file 1. Baseline survey. Pre-commencement survey questionnaire. [file 40337_2022_617_MOESM1_ESM.pdf]

ADDITIONAL FILE 1 BASELINE SURVEY

BASELINE SURVEY (pre-commencement)

1. Please enter the code for your FPS group
2. How would you describe the geographical location of your dietetic practice?
    a. Capital city
    b. Metropolitan centre (pop >100,000)
    c. Rural centre (10,000-99,000)
    d. Remote centre (5,000-9,999)
    e. Remote area (<5,000)
3. In what area are you currently employed?
    a. Public hospital service
    b. Public community health service
    c. Private hospital
    d. Private practice
    e. University clinic
    f. Public ED specialist service
    g. Non-government organisation
    h. Dietetic student
4. How many years of clinical dietetic experience do you have?
    a. <5years
    b. 5-10years
    c. >10years
5. What age range of eating disorder patients do you manage?
    a. Paediatric
    b. Adolescent
    c. Adult
6. In what settings do you treat eating disordered clients?
    a. Paediatric medical wards
    b. Adolescent medical wards
    c. Adult medical wards
    d. Adolescent mental health wards
    e. Adult mental health wards
    f. Medical outpatient clinics
    g. Mental health outpatient clinics
    h. Community mental health clinics
    i. Private practice clinics

    j. University clinics
    k. Non-government organisations
7. How many clients with eating disorders did you provide dietetic care for in the past 12months?
    a. <5 patients
    b. 5-15 patients
    c. >15 patients
8. Do you currently receive clinical/professional supervision/mentoring in the field of eating disorders?
9. Have you received ED-specific supervision in the past?
10. Do you feel that ED-specific clinical dietetic supervision is readily available?
11. Do you currently provide clinical/professional supervision to dietitians?
12. Please indicate whether you agree with the following statements (Likert scale)
    a. I feel confident applying evidence-based practice in the treatment of EDs
    b. I feel confident engaging/communicating with people with EDs
    c. I feel supported as a dietitian working in the field of EDs
13. How would you prefer to be supported to improve your skill in dietetic intervention for clients with eating disorders? Ranking scale.
    a. Facilitated peer group supervision (FPS)
    b. Individual clinical supervision
    c. Education sessions delivered by an ED specialist
    d. Online education modules
    e. Clearly documented guidelines/nutrition pathways
    f. Seminars/workshops
    g. Peer group supervision
14. Do you have any comments to make before we begin the Facilitated Peer Group Supervision Sessions?
