# Supplementary material for: Evaluation of a model of online, facilitated, peer group supervision for dietitians working in eating disorders
Source: J Eat Disord. 2022 Jul 4;10:93. doi: 10.1186/s40337-022-00617-7 (PMC9252553; doi:10.1186/s40337-022-00617-7)
Supplement: Supplementary file 2 — Additional file 2. Follow-up survey. 6 month follow-up questionnaire. [file 40337_2022_617_MOESM2_ESM.pdf]

ADDITIONAL FILE 2 FOLLOW UP SURVEY

FOLLOW-UP (6month) SURVEY

1. Please enter the code for your FPS group.
2. How would you describe the geographical location of your dietetic practice?
3. In what area are you currently employed?
4. How many years of clinical dietetic experience do you have?
5. What age range of eating disorder patients do you manage?
6. In what settings do you treat eating disordered clients?
7. How many clients with eating disorders did you provide dietetic care for in the past 12months?
8. Do you currently receive clinical/professional supervision/mentoring in the field of eating disorders in addition to attending QuEDS FPS?
9. Have you received ED-specific supervision in the past?
10. Do you feel that ED-specific clinical dietetic supervision is readily available?
11. Do you currently provide clinical/professional supervision to dietitians?
12. Please indicate your agreement with the following statements (Likert scale)
    a. I feel confident applying evidence-based practice in the treatment of EDs
    b. I feel confident engaging/communicating with people with EDs
    c. I feel supported as a dietitian working in the field of EDs
13. How would you prefer to be supported to improve your skill in dietetic intervention for clients with eating disorders? (ranking)
    a. Individual clinical supervision
    b. Facilitated peer group support (FPS)
    c. Education sessions delivered by a specialist
    d. Online education modules
    e. Clearly documented guidelines/nutrition pathways
    f. Seminars/workshops
    g. Peer group supervision
14. Please indicate if you agree or disagree with the following statements as a member of a QuEDS FPS group
    a. The group is too structured
    b. Members feel safe enough to expose their practice in the group setting
    c. Members are giving advice and other less than helpful responses
    d. Members feel equal to other members in the group
    e. There is sufficient time to meet the supervision needs of the group
    f. Some individuals are dominating, and others have become passive

g. Personalities or group dynamics are impacting on the quality of the supervision
h. Confidentiality of issues discussed is being maintained
i. Members feel criticised or demoralised
j. Members feel supported by the group
k. Members feel the group impacts positively on feelings of confidence
l. Facilitation of FPS impacts positively on group process

15. Now you have completed 6months of FPS do you have any feedback/comments to help us improve this model of professional support?
