# Supplementary material for: Evaluation of a model of online, facilitated, peer group supervision for dietitians working in eating disorders
Source: J Eat Disord. 2022 Jul 4;10:93. doi: 10.1186/s40337-022-00617-7 (PMC9252553; doi:10.1186/s40337-022-00617-7)
Supplement: Supplementary file 3 — Additional file 3. Learning and clinical practice survey. Questionnaire to determine clinical practice change secondary to participation in FPS. [file 40337_2022_617_MOESM3_ESM.pdf]

ADDITIONAL FILE 3 LCP SURVEY

LEARNING AND CLINICAL PRACTICE SURVEY

1. Which group are you currently a member? If you have transferred groups, please indicate your current group. If you are no longer participating, please indicate the group that you participated in.
2. How long have you (did you) participated in QuEDS FPS?
    a. <6months
    b. >6months
    c. >12months
3. Consider when you first joined QuEDS FPS. What did you hope to learn/gain from participating in the QuEDS FPS group?
    a. Knowledge of ED-specific evidence-based practice/guidelines
    b. Knowledge of ED-specific resources/tools
    c. Clinical knowledge of eating disorder presentations
    d. Knowledge of assessment/treatment of eating disorder cases
    e. ED-specific counselling skills
    f. Understanding of formulation of management of complex cases
    g. Confidence in ED-specific dietetic interventions
    h. Support from colleagues for you work in the ED arena
    i. Other, add comment below.
4. Did you have any expectations for FPS that you felt were NOT met? Please indicate below any learning areas that you felt were NOT adequately addressed.
    a. Knowledge of ED-specific evidence-based practice/guidelines
    b. Knowledge of ED-specific resources/tools
    c. Clinical knowledge of eating disorder presentations
    d. Knowledge of assessment/treatment of eating disorder cases
    e. ED-specific counselling skills
    f. Understanding of formulation of management of complex cases
    g. Confidence in ED-specific dietetic interventions
    h. Support from colleagues for you work in the ED arena
    i. Other, add comment below.
5. What have you gained from attending the FPS group? Please indicate your level of agreement with the following statements (Likert scale).
   "Participation in the QuEDS FPS group enabled me....."
    a. To become more confident in my clinical work
    b. To feel supported in my clinical work
    c. To meet ongoing professional development requirements

d. To increase my reflective practice
e. To increase my supervisory/mentoring skills
f. To cope better with stressors of working with ED clients
g. To better enjoy my work in the ED arena
h. To achieve more in my ED-specific clinical work

6. Has participation in QuEDS FPS changed your clinical practice? Please indicate your level of agreement with the following statements (Likert scale). If you wish to make further comments, please add to the text box below.
   "Participation in QuEDS FPS has changed my clinical practice within the ED arena as demonstrated by..."
   a. More appropriate implementation of evidence-based practice/guidelines
   b. Improved engagement with ED clients
   c. Application of ED-specific resources/tools
   d. Increased advocacy for appropriate care for ED clients
   e. Increased ability to provide dietetic intervention for complex ED cases
   f. Active engagement in ED-specific service development e.g., Local ED guidelines, quality projects, resource development, seeking more ED referrals
7. Do you believe QuEDS FPS model of clinical support should be continued and expanded? Please indicate your level of agreement with the following statements (Likert scale).
   a. I would recommend QuEDS FPS to other dietitians
   b. I would like to continue with QuEDS FPS
   c. I prefer the format of QuEDS FPS to standard Peer Group Supervision
   d. QuEDS FPS is a valuable adjunct to my clinical supervision/mentoring
   e. Participation in QuEDS FPS has led me to change my clinical practice
   f. The QuEDS FPS model would be appropriate for other clinical areas/disciplines
8. Are you still participating in QuEDS FPS?
9. If you are no longer participating in FPS, please indicate why?
   a. FPS did not meet my learning/support needs
   b. Workload commitments
   c. Position change
   d. Lack of support from operational lead
   e. Leave from position e.g., maternity
   f. Other reasons, please detail
10. Do you have any other feedback (positive or negative) re: your experience of QuEDS FPS?

(NB terminology has been updated from F-PGS to FPS)
