# Supplementary material for: Evaluation of a model of online, facilitated, peer group supervision for dietitians working in eating disorders
Source: J Eat Disord. 2022 Jul 4;10:93. doi: 10.1186/s40337-022-00617-7 (PMC9252553; doi:10.1186/s40337-022-00617-7)
Supplement: Supplementary file 4 — Additional file 4. Responses in Kirkpatrick levels. Responses according to the four levels for Kirkpatrick’s Model of Evaluation. [file 40337_2022_617_MOESM4_ESM.pdf]

ADDITIONAL FILE 4. Responses according to the four levels for Kirkpatrick's Model of Evaluation.

	Agree	Neutral	Disagree
**Kirkpatrick Level 1 - Reaction**			
The group is too structured	2 (5.4%)	4 (11%)	29 (78%)
Members feel safe enough to expose their practice in the group setting	35 (95%)	2 (5.4%)	-
Members are giving advice and other less than helpful responses	6 (16%)	2 (5.4%)	29 (78%)
Members feel equal to other members in the group	27 (73%)	9 (24%)	1 (2.7%)
There is sufficient time to meet the supervision needs of the group	26 (70%)	9 (24%)	2 (5.4%)
Some individuals are dominating, and others have become passive	1 (2.7%)	8 (22%)	28 (76%)
Personalities or group dynamics are impacting on the quality of the supervision (1 missing)	-	1 (2.7%)	35 (95%)
Confidentiality of issues discussed is being maintained	37 (100%)	-	-
Members feel criticised or demoralised	-	1 (2.7%)	36 (97%)
Members feel the group impacts positively on feelings of confidence	37 (100%)	-	-
Members feel supported in the group	35 (95%)	2 (5.4%)	-
Facilitation of FPS impacts positively on group process	36 (97%)	1 (2.7%)	-
Preferred method of clinical support - See body of results			
Plan to continue	47(96%)	2(4%)	
**Kirkpatrick Level 2 - Learning**			
Learning expectations/met or not met - See body of results –[see Additional file 5 learning expectations.pdf]			

I feel confident applying evidence-based practice in the treatment of EDs	33 (89%)	3 (8.1%)	2 (2.7%)
I feel confident engaging/communicating with people with EDs	35 (95%)	2 (5.4%)	
Participation in the FPS group enabled me to…			
To increase my clinical knowledge/skills	49 (98%)	1 (2%)	-
To increase my supervisory/mentoring skills	33 (67%)	14 (28%)	3 (6%)
To meet ongoing professional development requirements	42 (84%)	8 (16%)	-
**Kirkpatrick Level 3 – Behaviour/Implementation of Learnings**			
Participation in FPS has changed my clinical practice as evidenced by…			
More appropriate implementation of evidence-based practice/guidelines	44 (88%)	6 (12%)	-
Application of ED-specific resources/tools	45 (90%)	4 (11%)	-
Increased ability to provide dietetic intervention for complex ED cases	47 (94%)	2 (5.4%)	1 (2%)
Participation in the FPS group enabled me to…			
To increase my reflective practice	44 (88%)	6 (12%)	-
**Kirkpatrick Level 4 – Results/Broader Impacts**			
I feel supported as a dietitian working in the field of EDs	31 (84%)	4 (11%)	2 (5.4%)
Participation in the FPS group enabled me to…			
To become more confident in my clinical work	48 (96%)	2 (4%)	-
To feel supported in my clinical work	49 (98%)	1 (2%)	-
To cope better with stressors of working with ED clients	43 (86%)	5 (10%)	-

To better enjoy my work in the ED arena	42 (84%)	7 (14%)	1 (2%)
To achieve more in my ED-specific clinical work	47 (94%)	2 (4%)	1 (2%)
Participation in FPS has changed my clinical practice as evidenced by…			
Improved engagement with ED clients	44 (88%)	6 (12%)	-
Increased advocacy for appropriate care for ED clients	45 (90%)	5 (10%)	-
Active engagement in ED-specific service development	40 (80%)	9 (18%)	-
Participation in QuEDS FPS has led me to change my clinical practice	49 (98%)	1 (2%)	-
**Sustainability**			
I would like to continue with QuEDS FPS	48 (96%)	2 (4%)	-
I would recommend QuEDS FPS to other dietitians	50 (100%)	-	-
I prefer the format of QuEDS FPS to standard Peer Group Supervision	46 (92%)	4 (8%)	-
QuEDS FPS is a valuable adjunct to my clinical supervision/mentoring	49 (98%)	1 (2%)	-
The QuEDS FPS model would be appropriate for other clinical areas/disciplines	48 (96%)	2 (4%)	-
