# Supplementary material for: Evaluation of a model of online, facilitated, peer group supervision for dietitians working in eating disorders
Source: J Eat Disord. 2022 Jul 4;10:93. doi: 10.1186/s40337-022-00617-7 (PMC9252553; doi:10.1186/s40337-022-00617-7)
Supplement: Supplementary file 5 — Additional file 5. Learning expectations. Participants learning expectations of QuEDS FPS and unmet expectations. [file 40337_2022_617_MOESM5_ESM.pdf]

ADDITIONAL FILE 5 LEARNING EXPECTATIONS

Learning expectations as reported by eating disorder dietitians in the learning and clinical practice (LCP) survey (n=50)

	N	%	Unmet #
Knowledge of ED-specific evidence-based practice/guidelines	28	56%	4
Knowledge of ED-specific resources/tools	43	86%	3
Clinical knowledge of eating disorder presentations	29	58%	1
Knowledge of assessment/treatment of ED diagnoses	30	60%	4
ED-specific counselling skills	35	70%	6
Understanding of formulation of management plans for complex ED clients	28	56%	2
Confidence in ED-specific dietetic interventions	41	83%	1
Support from colleagues for your work in the ED arena	41	83%	0

number of participants with specific unmet learning expectations
